# Relationship of abdominal aortic calcification with lumbar vertebral volumetric bone mineral density assessed by quantitative computed tomography in maintenance hemodialysis patients

**DOI:** 10.1007/s11657-022-01059-z

**Published:** 2022-01-26

**Authors:** Tian-Yi Chen, Jie Yang, Li Zuo, Ling Wang, Li-Fang Wang

**Affiliations:** 1 Department of Renal Medicine, Beijing Jishuitan Hospital, No. 68, Hui-South Road, Hui- Longguan Town, Changping District 100096 Beijing, China; 2 Department of Renal Medicine, Peking University People’s Hospital, Beijing, China; 3 Department of Radiology, Beijing Jishuitan Hospital, Beijing, China; 4 Clinical Epidemiology Research Center, Beijing Jishuitan Hospital, Beijing, China

**Keywords:** Maintenance hemodialysis (MHD), Abdominal aortic calcification (AAC), Volumetric bone mineral density (vBMD), Quantitative computed tomography (QCT)

## Abstract

**Introduction:**

This cross-sectional study aimed to investigate the relationship between abdominal aortic calcification (AAC), which is a marker of vascular calcification, and volumetric bone mineral density (vBMD) by quantitative computed tomography (QCT) in maintenance hemodialysis (MHD) patients.

**Methods:**

All participants underwent lumbar vertebral vBMD measurement by QCT. Eight cross-sections were extracted sequentially and analyzed by ImageJ software to obtain the ratio of the calcified area to the abdominal aortic area (the calcification ratio). The AAC score was determined by the sum of the calcification ratios. The relationship between AAC and vBMD was analyzed using multivariate logistic regression.

**Results:**

Ninety MHD patients (58.89% male) with a mean age of 63.43 (standard deviation [SD] = 13.20) years were included in the study. AAC was present (AAC score > 0) in 93.33% of the patients. The 75th percentile of the AAC score corresponding to 119 was used as the cutoff point between the mild and severe groups. After full adjustment in the logistic model, AAC was found to be inversely associated with vBMD (odds ratio [OR], 0.970; 95% confidence interval [CI], 0.944 to 0.996; *P* = 0.025), and patients with osteoporosis had a significantly higher risk of severe AAC than those with normal bone mass (OR, 14.498; 95% CI, 1.507 to 139.486; *P* = 0.021). The independent inverse association was still stable after adjusting for variables measured at different time periods and using different cutoff points of the AAC score.

**Conclusion:**

There was an independent inverse association between AAC and vBMD, and osteoporosis was significantly associated with severe AAC in patients with MHD.

**Supplementary Information:**

The online version contains supplementary material available at 10.1007/s11657-022-01059-z.

## Introduction

Vascular calcification and osteoporosis are salient in maintenance hemodialysis (MHD) patients. The risk of cardiovascular disease (CVD) events is independently predicted by vascular calcification [1, 2]. CVD is present in more than 50% of patients undergoing dialysis, and the relative risk of death due to CVD events in MHD patients is reported to be 20 times higher than that in the general population [3]. Fragility fracture, a devastating clinical consequence of osteoporosis, leads to higher mortality and disability rates, as well as a greater healthcare burden. The overall relative risk for hip fracture is approximately 4 times higher for MHD patients than for individuals of the same sex in the general population [4, 5]. Vascular calcification and osteoporosis are age-related diseases and share some common risk factors [6]. Thus, it is necessary to clarify the association between them, which is useful for further investigating the underlying mechanisms and finding ways to stop the progression of these two diseases.

Prior studies have found a relationship between vascular calcification and osteoporosis in the general population [7], postmenopausal women [8], and diabetic patients [9]. There is increasing evidence that bone and vascular calcification share a common pathogenesis [6], and bone loss and vascular calcification often occur simultaneously. This contradictory deposition of calcium in bone and in the vasculature is commonly referred to as the “calcification paradox” or the bone-vascular axis [10]. Abnormalities in mineral homeostasis, which are common in MHD patients, accelerate the progression of vascular calcification and osteoporosis diagnosed according to bone mineral density (BMD); therefore, these two complications are more serious in MHD patients than in other populations. Some studies have reported an independent association between coronary artery calcification (CAC) and osteoporosis in MHD patients [11–13]. Abdominal aortic calcification (AAC) is a marker of vascular calcification, and recent studies have indicated that AAC has a high predictive value for the mortality of dialysis patients [14]. Compared to CAC, AAC can be assessed simultaneously during lumbar vertebral BMD testing, avoiding multiple tests and excessive exposure to X-rays. However, there are still few studies on AAC and BMD in MHD patients [15, 16]. In addition, there are some methodological limitations in these studies. Dual energy X-ray absorptiometry (DXA) and other X-ray radiographs are 2D methods for measuring areal BMD, which are confounded by degenerative bone changes and extraosseous calcification. Bone disease and vascular calcification are severe in MHD patients, reducing the accuracy of the measurement of these methods.

This study investigated the relationship between AAC and volumetric bone mineral density (vBMD) in MHD patients. Based on the bone-vascular axis hypothesis, we assumed that there was an inverse association between AAC and vBMD in patients with MHD. In view of the deficiency of DXA in measuring BMD, we used quantitative computed tomography (QCT) to measure the vBMD of lumbar vertebral trabecular bone. In contrast to DXA, QCT can avoid the overlap of tissue and distinguish between trabecular and cortical compartments of the lumbar spine. Moreover, using QCT images, eight cross-sections were extracted to quantitatively assess AAC. On this basis, the relationship between AAC and vBMD was analyzed by multivariate logistic regression.

## Methods

### Participants

**Fig. 1:**
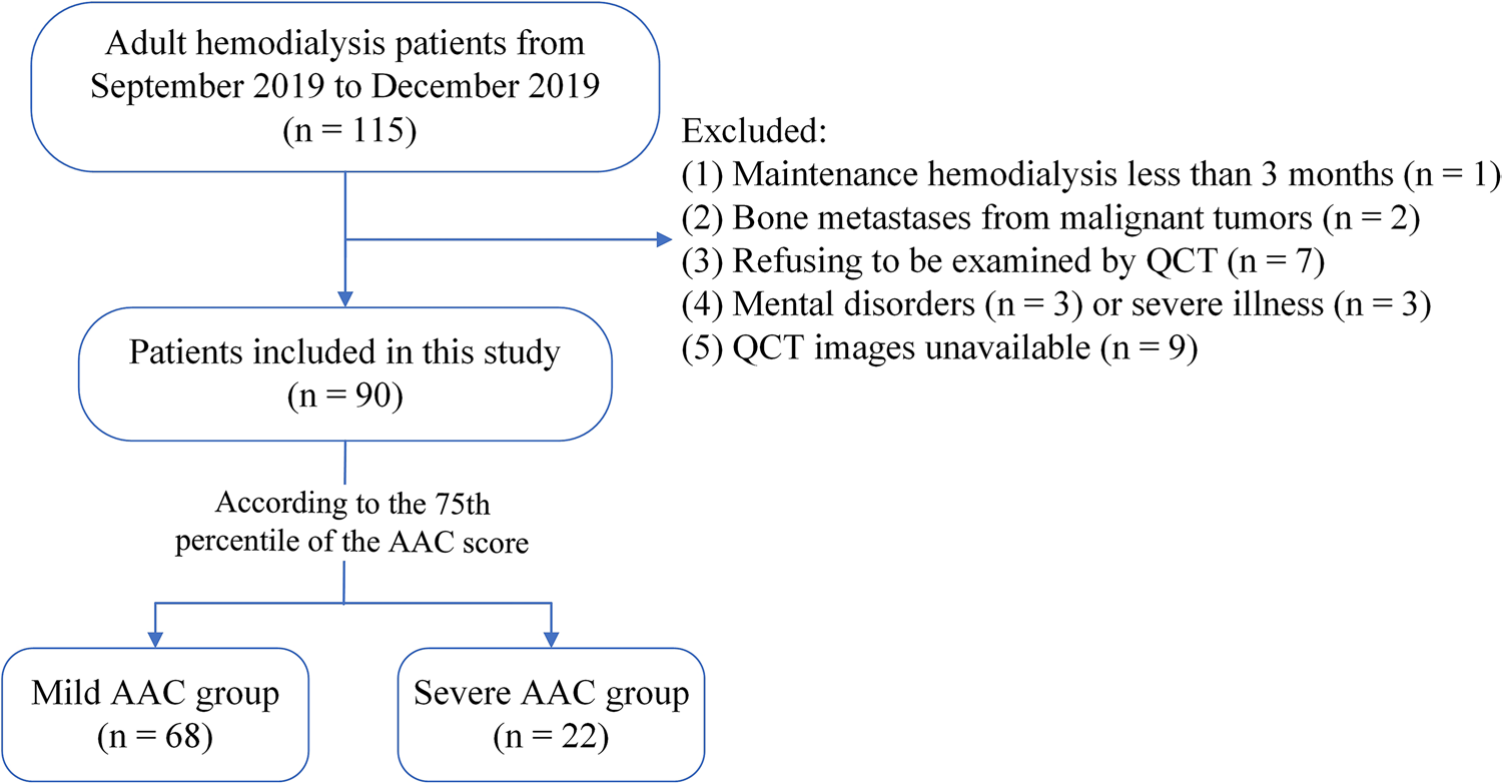
Flow diagram of participants throughout the study

A total of 90 adult MHD patients who underwent lumbar spine QCT scans were enrolled between September 2019 and December 2019 at the Department of Renal Medicine, Beijing Jishuitan Hospital, China. Among these patients, 2 underwent parathyroidectomy, 2 had hypothyroidism, 2 underwent renal transplantation, and some received glucocorticoid (GC) and/or immunosuppressant treatment. These conditions may affect AAC and vBMD. However, all patients who underwent parathyroidectomy suffered from recurrent hyperparathyroidism before QCT and received oral cinacalcet. Hypothyroid patients received regular levothyroxine treatment, and their thyrotropin and free thyroxine levels were normal at the whole time. Kidney transplant recipients had suffered from renal graft loss for several years and were on stable maintenance hemodialysis. GC and immunosuppressive agents have been withdrawn for many years in those who received these medications. According to relevant studies, the above situation may have little impact on AAC and vBMD [17–20]. As a consequence, patients with these conditions were included in this study. Finally, the exclusion criteria were as follows: (1) maintenance hemodialysis less than 3 months; (2) patients with bone metastases from malignant tumors; and (3) patients who were unable to cooperate with QCT examination. The flow diagram is displayed in Fig. 1.

The excluded patients were older (*P* = 0.033) and had lower albumin (*P* = 0.004) than the included patients because most patients who could not cooperate with QCT examination were old and seriously ill. There were no significant differences in the other variables. See the Appendix for all the parameters and analysis.

### Data collection for covariates

The demographic information and relevant clinical data were based on Beijing Jishuitan Hospital’s medical records and interviews with all participants. To calculate body mass index (BMI), height was measured within 3 months before QCT, and weight was expressed as dry weight. Patients with established coronary heart disease, diabetes mellitus, and peripheral arterial disease were considered to have coronary risk equivalents [21]. AAC and vBMD can be affected by the use of some medications, such as cinacalcet, calcitriol, calcium-containing phosphate binders, and non-calcium-containing phosphate binders. Therefore, information on the usage of these medications during the same period when patients underwent QCT was extracted from patients’ medical records.

Fasting blood samples were obtained before hemodialysis. All laboratory analyses, including intact parathyroid hormone (iPTH), 25-hydroxycholecalciferol (25-(OH)-D3), alkaline phosphatase (ALP), high-sensitivity C-reactive protein (hsCRP), ferritin, serum lipids and lipoproteins, minerals and electrolytes, and others, were measured at the central laboratory of Beijing Jishuitan Hospital. The parameter of hemodialysis adequacy, namely, Kt/V (urea), was calculated according to the Kidney Disease Outcomes Quality Initiative (KDOQI) [22].

### Measurement of lumbar vertebral volumetric bone mineral density (vBMD)

All of the lumbar vertebrae were scanned with a Toshiba CT scanner (Aquilion 64-slice, Toshiba Medical Systems Corporation, Tokyo, Japan). The following scanner settings were used: 120 kV, 125 mAs, slice thickness 1 mm, field-of-view 50 cm, matrix 512 × 512, and pitch 0.938. A QCT calibration phantom (Mindways Inc., Austin, TX, USA) was placed beneath the patients’ spine and scanned simultaneously. Images were analyzed using QCT Pro 5.0.3 software (Mindways Inc.). The vBMD values of the L2-4 vertebral body were measured separately. An elliptical region of interest (ROI) was placed in the central plane of the vertebral body, avoiding the cortical bone of the vertebrae and the vertebral veins. Fractured vertebrae were excluded. Then, the mean vBMD value of L2-4 was calculated as the patient’s vBMD parameter. The criterion suggested by the latest Chinese expert consensus [23] was used to stratify vBMD in this study. For spinal trabecular vBMD, the thresholds were > 120 mg/cm^3^ for normal, 80 mg/cm^3^ ≤ vBMD ≤ 120 mg/cm^3^ for osteopenia, and < 80 mg/cm^3^ for osteoporosis, which was the same criterion used by the American College of Radiology in 2008 [24].

### Measurement of abdominal aortic calcification (AAC)

**Fig. 2:**
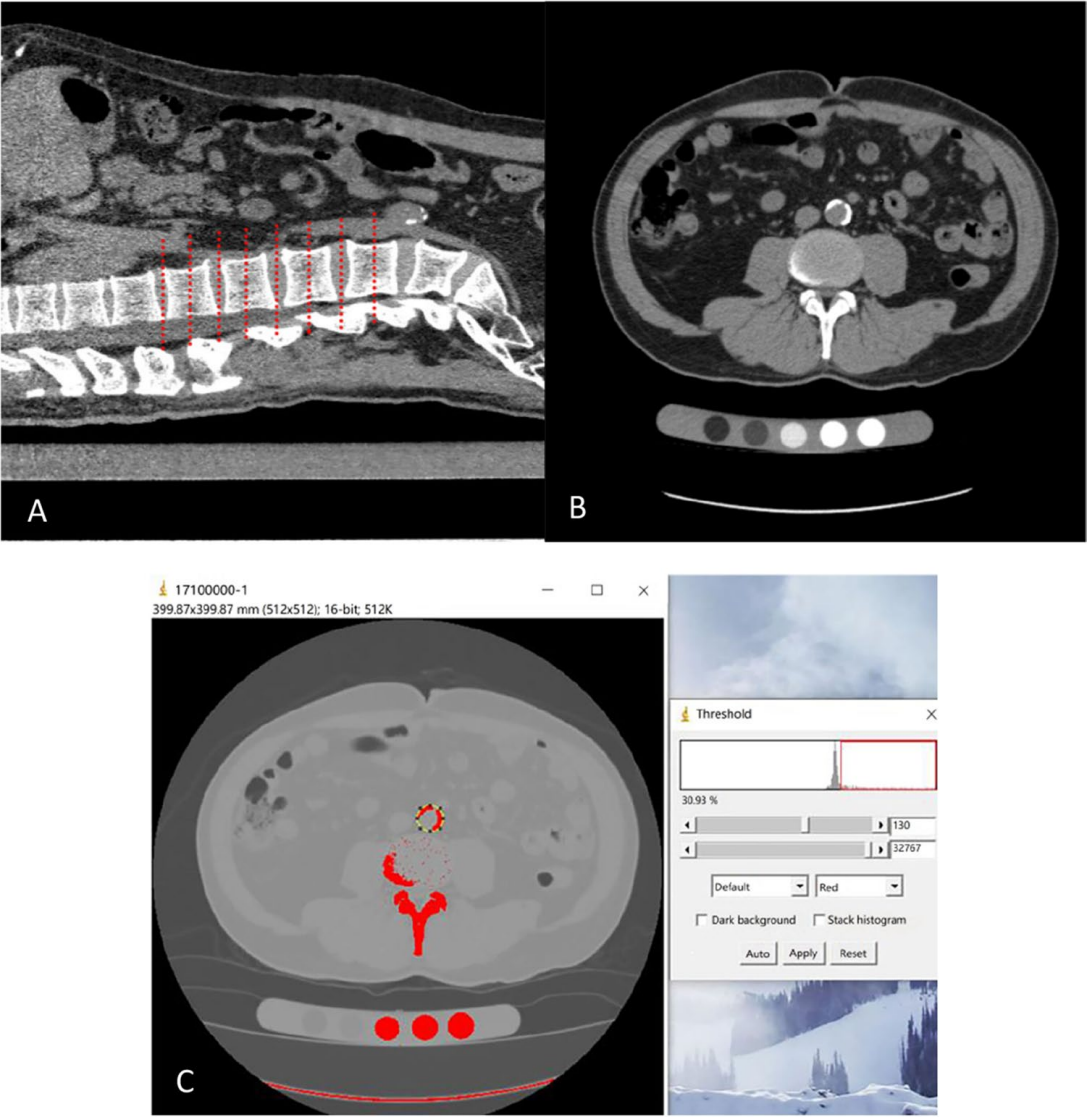
Measurement of abdominal aortic calcification (AAC). **A**: The sagittal QCT image of the lumbar spine shows 8 cross-sections to be measured (red dotted line). **B**: Scanning image of the central plane of the L3/L4 intervertebral discs. **C**: The measurement process of panel B: the yellow dotted circle is the area of the abdominal aorta, within which the red area is calcified, and the ratio of the two areas is 30.93%, as shown on the right

After the CT scan, raw data were transmitted to the workstation and reconstructed to a slice thickness of 3 mm. Starting from the T12/L1 intervertebral discs, 8 cross-sections from each central plane of the intervertebral disc and the vertebral body between T12 and L4 were extracted sequentially. Each cross-sectional image was then analyzed by ImageJ (National Institutes of Health, Bethesda, MD, USA) to measure the area of calcification and the cross-sectional area of the abdominal aorta. The percentage of the calcified area was obtained (Fig. 2). The AAC score was calculated as the sum of the calcification percentages of 8 cross-sections multiplied by 100. Specifically, the area of the abdominal aorta was delineated, and then, the lower limit of the CT threshold was adjusted to 130 Hounsfield units according to Agatston’s study [25]. ImageJ automatically generated the calcification ratio, which was the ratio of the calcified area to the abdominal aortic area at this cross-section. Finally, the sum of the calcification ratios of 8 cross-sections was multiplied by 100, which was the AAC score.

### Statistical analysis

Data are expressed as the median (interquartile range) or mean ± standard deviation (SD) for continuous variables and as the number (percentage) for discrete variables. In this study, univariate analysis and multivariate analysis were used. In univariate analysis, the 75th percentile (P75) of the AAC score was used as the cutoff point between the mild AAC and severe AAC groups in the main analyses. Group differences were tested using Student’s *t* test or the Mann–Whitney *U* test, depending on the distribution of the continuous variables, and using the Chi-squared or Fisher’s exact test for categorical variables. Based on vBMD stratification, patients were divided into three groups (normal, osteopenia, and osteoporosis). One-way analysis of variance (ANOVA) was applied to compare AAC scores among these three groups. In multivariate analysis, since the AAC score, which was the dependent variable, was non-normally distributed, the 75th percentile (P75) of the AAC score was used to divide participants into two groups, and then multivariate logistic regression models were performed to determine the association of AAC score with continuous vBMD and vBMD stratification. All variables with *P* < 0.1 in the univariate analysis were included in the multivariate regression model. Model 1 was unadjusted. Model 2 was adjusted for age, dialysis vintage, and primary disease. Compared with Model 2, Model 3 was additionally adjusted for phosphate, iPTH, Kt/V, and ferritin.

The main objective of this study was to investigate the relationship between AAC and vBMD in MHD patients. Therefore, sensitivity analyses were performed to check the stability of the results using the same statistical method. MHD patients were frequently monitored for Hb concentration, iPTH, minerals, and electrolytes, and these laboratory parameters fluctuated greatly under individual factors of patients and treatment intervention. Sensitivity analyses using the 6-month mean laboratory parameters (the mean value of each parameter was determined using all measurements performed during the 6 months prior to QCT) were conducted in univariate analysis and multivariate analysis. Other sensitivity analyses were conducted by selecting different cutoff points (P70, P65, P60) of the AAC score (see Appendix). Statistical significance was defined as a *P* value less than 0.05 (two-tailed). Analyses were conducted using SPSS 24.0 (IBM Corporation, Chicago, IL, USA).

## Results

### Clinical and biochemical characteristics

Ninety patients (58.89% male) with a mean age of 63.43 (SD = 13.20) years were included in the study. The mean dialysis vintage was 69.78 (SD = 48.30) months, and diabetic kidney disease was the most common reason for dialysis (37.78%). The AAC score ranged from 0 to 247, with a median score of 54.00 (18.00, 119.00), and AAC was present (AAC score > 0) in 93.33% of the patients. vBMD was distributed in a range of 29 mg/cm^3^–221 mg/cm^3^, and the mean was 104.22 mg/cm^3^ (SD = 39.44). The numbers of patients with osteoporosis, osteopenia, and normal bone mass were 27 (30%), 31 (34.44%), and 32 (35.56%), respectively.

Patients were divided into two groups according to the 75th percentile of the AAC score corresponding to 119, namely, a mild AAC group (≤ 119) and a severe AAC group (> 119). In the severe AAC group, the proportion of osteoporosis was the highest (54.54%) compared with the proportion of osteopenia (31.82%) and normal bone mass (13.64%). In the mild AAC group, the proportion of osteoporosis was the lowest (22.06%), the proportion of osteopenia was 35.29%, and the proportion of normal bone mass was the highest (42.65%). Patients in the severe AAC group were older; had a longer duration of dialysis, a lower proportion of diabetic kidney disease and a higher proportion of chronic glomerulonephritis; and exhibited lower vBMD but higher phosphorus, Kt/V, and ferritin levels than those in the mild AAC group. These variables differed significantly between the two groups (*P* < 0.05) (Table 1).

**Table 1:** Clinical and biochemical characteristics of patients divided into the mild group and severe group by the AAC score

Variables	All patients (***n*** = 90)	Mild AAC group (***n*** = 68)	Severe AAC group (***n*** = 22)	*P* values
Age, years	63.43 ± 13.20	61.25 ± 13.29	70.18 ± 10.59	**0.005**
Male (%)	53 (58.89)	43 (63.24)	10 (45.45)	0.212
Dialysis vintage, months	69.78 ± 48.30	60.88 ± 43.44	97.27 ± 53.07	**0.002**
BMI, kg/m^2^	23.84 ± 3.79	23.78 ± 3.89	24.01 ± 3.56	0.806
Current smoker (%)	18 (20.00)	15 (22.06)	3 (13.64)	0.544
Coronary risk equivalents (%)	49 (54.44)	35 (51.47)	14 (63.64)	0.339
Primary disease				
DKD (%)	34 (37.78)	30 (44.12)	4 (18.18)	**0.042**
CG (%)	22 (24.44)	13 (19.12)	9 (40.91)	**0.049**
HRD (%)	13 (14.44)	9 (13.24)	4 (18.18)	0.728
Other (%)	21 (23.33)	16 (23.53)	5 (22.73)	1.000
Medication use (during QCT)^a^				
Cinacalcet (%)	28 (31.11)	19 (27.94)	9 (40.91)	0.253
Calcitriol (%)	27 (30.00)	20 (29.14)	7 (31.82)	0.830
Calcium-containing phosphate binders (%)	61 (67.78)	46 (67.65)	15 (68.18)	0.963
Non-calcium-containing phosphate binders (%)	42 (46.67)	31 (45.59)	11 (50.00)	0.718
AAC score	54.00 (18.00, 119.00)	32.00 (10.50, 75.00)	154.00 (131.75, 179.75)	** < 0.001**
vBMD, mg/cm^3^	104.22 ± 39.44	111.82 ± 38.66	80.73 ± 32.50	**0.001**
vBMD stratification				
Normal (%)	32 (35.56)	29 (42.65)	3 (13.64)	**0.020**
Osteopenia (%)	31 (34.44)	24 (35.29)	7 (31.82)	0.490
Osteoporosis (%)	27 (30)	15 (22.06)	12 (54.54)	**0.007**
Corrected calcium, mmol/L	2.23 ± 0.17	2.22 ± 0.17	2.26 ± 0.16	0.304
Phosphate, mmol/L	1.76 ± 0.50	1.7 ± 0.47	1.96 ± 0.53	**0.032**
iPTH, pg/ml	201 (127.6, 319.68)	178 (116.05, 306.38)	243.9 (163.75, 500.3)	0.086
Hemoglobin, g/L	117.42 ± 9.98	116.71 ± 9.49	119.64 ± 11.32	0.233
Albumin, g/L	39.36 ± 2.45	39.37 ± 2.30	39.35 ± 2.91	0.969
TG, mmol/L	1.96 ± 1.27	1.90 ± 1.32	2.13 ± 1.11	0.459
TC, mmol/L	3.73 ± 0.84	3.67 ± 0.81	3.91 ± 0.91	0.253
LDL-C, mmol/L	1.94 ± 0.67	1.90 ± 0.68	2.06 ± 0.65	0.343
CO_2_CP, %	49.93 ± 5.83	49.75 ± 5.69	50.50 ± 6.33	0.603
ALP, IU/L	64 (54, 78)	64 (55, 77.50)	64.50 (50.75, 90.25)	0.749
Kt/V	1.40 ± 0.28	1.36 ± 0.28	1.51 ± 0.24	**0.034**
hsCRP, mg/L	2.02 (0.89, 5.95)	1.80 (0.80, 5.43)	3.70 (0.98, 12.65)	0.197
Ferritin, ng/ml	265.87 (222.56, 358.52)	250.01 (200.41, 329.16)	325.50 (249.57, 468.83)	**0.016**
25-(OH)-D3, ng/ml	16.10 ± 7.10	15.65 ± 6.57	17.50 ± 8.53	0.292

The bold data represent *P* values are less than 0.05

Abbreviations: *BMI* body mass index, *AAC* score abdominal aortic calcification score, *DKD* diabetic kidney disease, *CG* chronic glomerulonephritis, *HRD* hypertensive renal damage, *vBMD* volumetric bone mineral density, iPTH intact parathyroid hormone, *TG* triglyceride, *TC* total cholesterol, *LDL-C* low-density lipoprotein cholesterol, *CO*_*2*_*CP* carbon dioxide combining power, *ALP* alkaline phosphatase, *hsCRP* high-sensitivity C-reactive protein, *25-(OH)-D3* 25-hydroxycholecalciferol

^**a**^Only the medications in the table were included in this study. Glucocorticoids, immunosuppressants, systemic anticoagulants, and bisphosphonates that may affect AAC and vBMD were not included. The reason was that no patients received these medications during the same period of QCT

### Difference of AAC score among different vBMD stratification

**Fig. 3:**
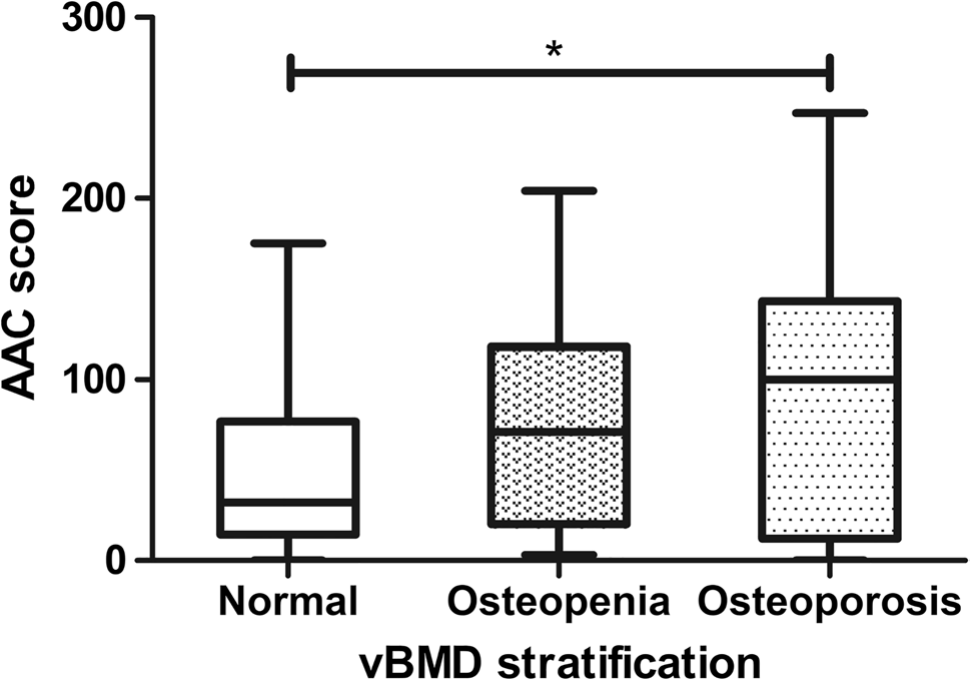
The AAC score among different vBMD stratification

The median AAC scores of the three groups (normal, osteopenia, and osteoporosis) were 32.00 (14.25, 76.75), 71.00 (20.00, 118.00), and 100.00 (12.00, 143.00), respectively. The lower the bone mass, the higher the AAC score. Moreover, there was a significant difference in AAC score between patients in the normal bone mass group and osteoporosis patients (*P* = 0.018) (Fig. 3).

### Association of AAC with continuous vBMD and vBMD stratification

On the basis of univariate analysis, a multivariate logistic regression model was applied to further verify the relationship between AAC and vBMD. The results suggested that AAC was inversely correlated with vBMD. In other words, vBMD was lower in patients with severe AAC. The inverse correlation remained significant (OR, 0.970; 95% CI, 0.944 to 0.996; *P* = 0.025) after adjusting for age, sex, dialysis vintage and primary disease, phosphate, iPTH, Kt/V, and ferritin. Moreover, the relationship between AAC and vBMD stratification was analyzed (Table 2). Whether or not these covariates were adjusted, compared with patients in the normal bone mass group, osteoporosis patients were significantly more susceptible to severe AAC (*P* < 0.05), while osteopenia patients showed a trend to suffer from severe AAC, which was not statistically significant.

**Table 2:** Association of AAC with continuous vBMD and vBMD stratification in logistic models

AAC degree	Continuous vBMD			vBMD stratification						
	*B*	OR (95% CI)	*P* values	Normal^a^	Osteopenia			Osteoporosis		
					*B*	OR (95% CI)	*P* values	*B*	OR (95% CI)	*P* values
Model 1	− 0.025	0.975 (0.960, 0.991)	**0.002**	Ref	1.037	2.819 (0.657, 12.100)	0.163	2.046	7.733 (1.887, 31.687)	**0.004**
Model 2	− 0.026	0.974 (0.953, 0.996)	**0.019**	Ref	1.688	5.406 (0.872, 33.503)	0.070	2.466	11.771 (1.738, 79.703)	**0.012**
Model 3	− 0.030	0.970 (0.945, 0.996)	**0.024**	Ref	1.924	6.845 (0.889, 52.681)	0.065	2.582	12.524 (1.450, 108.139)	**0.022**

The bold data represent *P* values are less than 0.05

Model 1 Unadjusted; Model 2 Adjusted for age, dialysis vintage, and primary disease; Model 3 Adjusted for age, dialysis vintage, and primary disease, phosphate, iPTH, Kt/V, and ferritin.

^a^The normal bone mass group was regarded as the reference group.

## Discussion

This study measured the AAC and vBMD of vertebral trabecular bone in the same scan using QCT and assessed their association in MHD patients. An inverse relationship between AAC and vBMD was observed, which was stable even after adjusting for multiple covariates and selecting different grouping cutoff points of the AAC score. We demonstrated that low vBMD, especially osteoporosis, was significantly associated with severe AAC in MHD patients. This result suggests that CVD, predicted by vascular calcification, should be monitored closely in clinical practice for MHD patients with osteoporosis.

Vascular calcification is not a passive deposition of hydroxyapatite but an active cell-regulated osteogenic process [26]. FGF-23 (fibroblast growth factor 23), fetuin-A, matrix Gla protein, osteoprotein, and so on play an important role in vascular calcification; at the same time, these factors are also crucial in bone remodeling. There may be an interweaving mechanism between bone metabolism and vascular calcification [27]. Several clinical studies have shown that bone demineralization and vascular mineralization often go hand in hand [8, 9]. All these results indicated the existence of the bone-vascular axis. However, the results of studies on the relationship between AAC and vBMD in the general population were somewhat controversial. Two representative studies indicated an inverse relationship between these two measures [28, 29]. In addition, the China Action on Spine and Hip Status study (CASH) demonstrated that the association between AAC prevalence and vBMD was significant only in men [7]. Moreover, neither a study in Rochester nor a study of women in South Korea found that AAC correlated with vBMD [30, 31]. The current study found that vascular calcification in MHD patients had a stable independent inverse correlation with vBMD. An independent inverse association was also reported by two other studies on patients with end-stage renal disease (ESRD) [15, 16]. The consistency of these results among ESRD patients indicated that the correlation between AAC and vBMD in these patients may be stronger than that in the general population, suggesting the presence of a bone-vascular axis in these patients.

The relationship between vascular calcification and vBMD showed greater significance in MHD patients, which may be related to internal environment disorders caused by uremia. (1) Hyperphosphatemia is common and prominent in MHD patients and plays a key role in vascular calcification and osteoporosis. Higher serum phosphorus levels aggravate CAC. Each 1 mg/dl increase in phosphorus imparted odds ratios for CAC of 1.61 (incidence) and 1.54 (prevalence), risks comparable to traditional CVD risk factors [32]. In a previous experimental study, a rat model of 7/8 nephrectomy fed with high-phosphorus diet was used, and after 20 weeks, the rats showed a significant increase in serum phosphorus and parathyroid hormone (PTH), together with aortic calcification and a decrease in bone mass [33]. An in vitro experiment demonstrated that human aortic smooth muscle cells cultured in individuals with hyperphosphatemia (> 1.4 mmol/l) showed dose-dependent increases in mineral deposition, but in normal physiological levels of inorganic phosphate did not mineralize [34]. Hyperphosphatemia promotes and triggers the progression of vascular calcification by inducing VSMC apoptosis, leading to the transdifferentiation of VSMCs to osteoblasts, elevating FGF23 levels, and decreasing Klotho expression [35, 36]. Increased serum phosphate is known to affect bone metabolism directly and indirectly through the development of adaptive hormonal mechanisms aimed at preventing hyperphosphatemia, such as the increase in PTH and FGF23 and the reduction in calcitriol [37]. (2) Chronic inflammation, oxidative stress, and secondary hyperparathyroidism, which are also the main manifestations of internal environmental disorders in MHD patients, have opposite effects on bone and vascular calcification. Runt-related transcription factor 2 (Runx2), an osteoblast-specific transcription factor, plays a crucial role in promoting osteogenic differentiation of VSMCs, which leads to medial arterial calcification (MAC) in MHD patients [38]. Inflammation, oxidative stress, and high serum PTH levels upregulate Runx2 expression in VSMCs, thereby increasing matrix mineralization and the production of bone-related proteins [38–40]. In terms of bone metabolism, proinflammatory cytokines produced by activated macrophages and lymphocytes promote the expression of receptor activator of nuclear factor κ-B ligand (RANKL) in osteoblasts. RANKL recognizes its receptor, RANK, on the osteoclast surface and stimulates osteoclast formation. Activated osteoclasts cause bone resorption [41, 42]. Oxidative stress is considered a cause of osteoporosis. Reactive oxygen species (ROS) induce the apoptosis of osteoblasts and osteocytes, favor osteoclastogenesis, and inhibit mineralization and osteogenesis [43]. High serum PTH levels accelerate bone turnover in MHD patients. Furthermore, osteoclast activity overcomes osteoblast activity in high-turnover bone disease. Hyperparathyroidism leads to a net loss of bone mass caused by excessive stimulation of bone resorption [44].

A large-sample cross-sectional study in China used QCT scans to demonstrate that the prevalence of AAC was 30.5% (female) and 37.6% (male) in the general population aged 61.4–62.7 years [7]. Recent studies have shown that 49–60.7% of ESRD patients had prevalent AAC using X-ray [14, 16]. The prevalence of AAC in this study was 93.33%, which was higher than that in the general population because MHD patients are more susceptible to vascular calcification. The prevalence of AAC in this study was also higher than that in similar studies on patients with ESRD, mainly because of detection instruments. CT is more sensitive to small deposits of calcium than X-ray radiography. A meta-analysis showed that the prevalence of AAC in patients with dialysis was significantly higher in the 6 studies using CT (84.9%; 95% CI, 78.0 to 91.7%) than in the 37 studies using X-ray radiography (65.2%; 95% CI, 59.3 to 71.1%) [45].

Previous studies have formed a consensus that diabetes mellitus (DM) is a risk factor for vascular calcification [46–48]. In the present study, the univariate analysis suggested a lower proportion of severe AAC in patients with diabetic kidney disease (DKD) and a higher proportion of severe AAC in those with chronic glomerulonephritis (CG), which seemed to be inconsistent with the consensus. This may be due to differences in the dialysis vintage among patients, with an average of 49 months for patients with DKD and 100 months for patients with CG. Long dialysis vintage means that patients have long-term uremia status and mineral metabolism disorders, which are the trigger and aggravation factors for vascular calcification in MHD patients [36]. After full adjustment, only two variables, vBMD and dialysis vintage, were found to be independently correlated with AAC, while no significant association was indicated between primary disease and AAC. However, this finding does not deny the impact of DM on vascular calcification in MHD patients. Patients with DKD and those with CG were then matched according to the dialysis vintage. When the mean dialysis vintage was 49 months, the average AAC score of patients with CG was 48.4, while that of patients with DKD was 63.6. The results showed that DM also played a role in the progression of vascular calcification in MHD patients, which was consistent with the consensus. In summary, compared with the primary disease, dialysis vintage had a greater impact on AAC.

This study has some limitations. First, because of the cross-sectional design of this study, we could not infer the causal association between AAC and vBMD. Second, although the method used in this study to measure AAC scores has been used by Nakayama et al. [49], no studies have evaluated the accuracy of this method.

## Conclusions

This study indicated that there was an inverse stable relationship between AAC and vBMD. Osteoporosis was significantly associated with severe AAC in patients with MHD. Therefore, MHD patients with osteoporosis may have a higher risk of CVD, and intensive cardiovascular disease surveillance should be performed for MHD patients with low vBMD. A large prospective cohort study should be conducted to further clarify the causal relationship between AAC and vBMD. The mechanism of the bone-vascular axis needs to be elucidated to help inhibit the progression of vascular calcification and osteoporosis together in clinical practice.

## Supplementary Information

Below is the link to the electronic supplementary material.

Supplementary file1 (DOCX 75 KB)

## Data Availability

The data used during the current study are available from the corresponding author on reasonable request.
