# Supplementary material for: Relationship of abdominal aortic calcification with lumbar vertebral volumetric bone mineral density assessed by quantitative computed tomography in maintenance hemodialysis patients
Source: Arch Osteoporos. 2022 Jan 26;17(1):24. doi: 10.1007/s11657-022-01059-z (PMC8791896; doi:10.1007/s11657-022-01059-z)
Supplement: Supplementary file 1 — Supplementary file1 (DOCX 75 KB) [file 11657_2022_1059_MOESM1_ESM.docx]

**Supplementary Appendix**

**Relationship of** **abdominal aortic calcification with lumbar vertebral volumetric bone mineral density assessed by quantitative computed tomography in maintenance hemodialysis patients**

Tian-Yi Chen ^1^, Jie Yang ^1^, Li Zuo ^2^, Ling Wang ^3^, Li-Fang Wang ^4^

^1^ Department of Renal Medicine, Beijing Jishuitan Hospital, Beijing, China

^2^ Department of Renal Medicine, Peking University People’s Hospital, Beijing, China

^3^ Department of Radiology, Beijing Jishuitan Hospital, Beijing, China

^4^ Clinical Epidemiology Research Center, Beijing Jishuitan Hospital, Beijing, China

**Corresponding author**

Tian-Yi Chen

Department of Renal Medicine, Beijing Jishuitan Hospital, No. 68, Hui-South Road, Hui-Longguan Town, Changping District, Beijing 100096, China

Email: [chentianyi219@163.com](mailto:chentianyi219@163.com)

**1. Clinical and biochemical characteristics of the included and excluded patients**

The comparison of demographic information and biochemical parameters between the included and excluded patients is shown in Table 1. The analysis of the results revealed that the excluded patients were older (*P* = 0.033) and had lower albumin (*P* = 0.004) than the included patients, with no significant differences in the other variables. (1) Six of the 25 excluded patients were unable to undergo quantitative computed tomography (QCT) examination due to severe illness or mental disorder, and the mean age of these patients was 78.33 (SD = 10.78) years. The mean age of the remaining 19 excluded patients was 67.11 (SD = 12.71) years after excluding these 6 patients, and there was no statistically significant difference with the included patients (*P* = 0.270). (2) Two of the excluded patients contracted severe lower limb gangrene and infection, three of the excluded patients had mental abnormalities and poor feeding, and another two patients of the excluded patients suffered from bone metastasis of breast cancer and colon cancer, which caused a mass of consumption and asthenia. Therefore, there was a significant difference in albumin between the included patients and the excluded patients. After excluding these 7 patients, the average value of albumin in the remaining 18 excluded patients was 38.63 (SD = 2.35) g/L, with no statistically significant difference from the included patients (*P* = 0.244). In summary, after excluding these special cases, there was no significant difference between the included patients and the excluded patients.

**Supplemental Table 1** Clinical and biochemical characteristics of the included and excluded patients

| Variables | Included patients  (n = 90) | Excluded patients  (n = 25) | *P*-Values |
| --- | --- | --- | --- |
| Age, years | 63.43 ± 13.20 | 69.80 ± 12.60 | **0.033** |
| Male (%) | 53 (58.89) | 15 (60.00) | 0.920 |
| Dialysis vintage, months | 69.78 ± 48.30 | 57.60 ± 43.61 | 0.258 |
| BMI, kg/m^2^ | 23.84 ± 3.79 | 23.76 ± 3.89 | 0.932 |
| Current smoker (%) | 18 (20.00) | 4 (16.00) | 0.779 |
| Coronary risk equivalents (%) | 49 (54.44) | 16 (64.00) | 0.394 |
| Primary disease |  |  |  |
| DKD (%) | 34 (37.78) | 11 (44.00) | 0.573 |
| CG (%) | 22 (24.44) | 4 (16.00) | 0.372 |
| HRD (%) | 13 (14.44) | 4 (16.00) | 1.000 |
| Other (%) | 21 (23.33) | 6 (24.00) | 0.945 |
| Corrected calcium, mmol/L | 2.23 ± 0.17 | 2.21 ± 0.18 | 0.311 |
| Phosphate, mmol/L | 1.76 ± 0.50 | 1.69 ± 0.51 | 0.517 |
| iPTH, pg/ml | 201 (127.6, 319.68) | 197.4 (104.3, 319.55) | 0.776 |
| Hemoglobin, g/L | 117.42 ± 9.98 | 114.12 ± 14.70 | 0.193 |
| Albumin, g/L | 39.36 ± 2.45 | 37.56 ± 3.55 | **0.004** |
| TG, mmol/L | 1.96 ± 1.27 | 1.58 ± 0.68 | 0.396 |
| TC, mmol/L | 3.73 ± 0.84 | 3.85 ± 1.19 | 0.765 |
| LDL-C, mmol/L | 1.94 ± 0.67 | 2.16 ± 0.78 | 0.149 |
| CO_2_CP, % | 49.93 ± 5.83 | 49.16 ± 6.89 | 0.574 |
| ALP, IU/L | 64 (54, 78) | 70 (57.5, 87.5) | 0.252 |
| Kt/V | 1.40 ± 0.28 | 1.29 ± 0.25 | 0.085 |
| hsCRP, mg/L | 2.02 (0.89, 5.95) | 3.38 (2.07, 10.97) | 0.058 |
| Ferritin, ng/ml | 301.99 ± 170.39 | 273.58 ± 253.03 | 0.512 |
| 25-(OH)-D3, ng/ml | 16.10 ± 7.10 | 14.70 ± 5.89 | 0.369 |

Abbreviations: BMI: body mass index; DKD: diabetic kidney disease; CG: chronic glomerulonephritis; HRD: hypertensive renal damage; iPTH: intact parathyroid hormone; TG: triglyceride; TC: total cholesterol; LDL-C: low-density lipoprotein cholesterol; CO_2_CP: carbon dioxide combining power; ALP: alkaline phosphatase; hsCRP: high-sensitivity C-reactive protein; 25-(OH)-D3: 25-hydroxycholecalciferol

**2. Sensitivity analyses**

**2.1 Using 6-month mean laboratory parameters**

The stability of the study was checked using the 6-month mean laboratory parameters. The severe AAC group had significantly higher age, dialysis vintage, proportion of chronic glomerulonephritis and ferritin and lower vBMD than the mild AAC group (Supplemental Table 2). Unadjusted and adjusted models for AAC with continuous vBMD and vBMD stratification are presented in Supplemental Table 3. Consistent with the main analyses, there was an independent inverse correlation between AAC and continuous vBMD. The relationship between AAC and vBMD stratification had some differences. In Model 3, both osteopenia patients (OR, 10.648; 95% CI, 1.318 to 86.031; *P* = 0.026) and osteoporosis patients (OR, 15.796; 95% CI, 1.697 to 147.052; *P* = 0.015) had a higher risk of severe AAC than those in the normal bone mass group.

**Supplemental Table 2** Clinical and biochemical characteristics of patients divided into the mild group and severe group by AAC score (the 6-month mean laboratory parameters)

| Variables | All patients  (n = 86)^a^ | Mild AAC group  (n = 65)^b^ | Severe AAC group  (n = 21)^b^ | *P*-Values |
| --- | --- | --- | --- | --- |
| Age, years | 63.59 ± 13.33 | 61.51 ± 13.47 | 70.05 ± 10.83 | **0.010** |
| Male (%) | 51 (59.30) | 41 (63.08) | 10 (47.62) | 0.210 |
| Dialysis vintage, months | 71.70 ± 47.92 | 65.54 ± 47.69 | 90.76 ± 44.47 | **0.035** |
| BMI, kg/m^2^ | 23.72 ± 3.75 | 23.57 ± 3.83 | 24.19 ± 3.55 | 0.517 |
| Current smoker (%) | 16 (18.60) | 13 (20.00) | 3 (14.29) | 0.751 |
| Coronary risk equivalents (%) | 47 (54.65) | 33 (50.77) | 14 (66.67) | 0.203 |
| Primary disease |  |  |  |  |
| DKD (%) | 30 (34.88) | 26 (40.00) | 4 (19.05) | 0.080 |
| CG (%) | 22 (25.58) | 13 (20.00) | 9 (42.86) | **0.037** |
| HRD (%) | 13 (15.12) | 9 (13.85) | 4 (19.05) | 0.726 |
| Other (%) | 21 (24.42) | 17 (26.15) | 4 (19.05) | 0.510 |
| Medication use^c^ |  |  |  |  |
| Cinacalcet (%) | 28 (32.56) | 19 (29.23) | 9 (42.86) | 0.333 |
| Calcitriol (%) | 27 (31.40) | 20 (30.77) | 7 (33.33) | 0.960 |
| Calcium-containing phosphate binders (%) | 59 (68.60) | 44 (67.69) | 15 (71.43) | 0.960 |
| Non-calcium-containing phosphate binders (%) | 39 (45.35) | 28 (43.08) | 11 (52.38) | 0.611 |
| AAC score | 54.00 (17.25, 123.25) | 33.00 (9.50, 76.50) | 157.00 (134.00, 184.50) | **<0.001** |
| vBMD, mg/cm^3^ | 104.27 ± 38.69 | 111.43 ± 37.97 | 82.10 ± 32.65 | **0.002** |
| vBMD stratification |  |  |  |  |
| Normal (%) | 30 (34.88) | 27 (41.54) | 3 (14.29) | **0.023** |
| Osteopenia (%) | 31 (36.05) | 24 (36.92) | 7 (33.33) | 0.766 |
| Osteoporosis (%) | 25 (29.07) | 14 (21.54) | 11 (52.38) | **0.007** |
| Corrected calcium, mmol/L | 2.24 ± 0.15 | 2.23 ± 0.14 | 2.28 ± 0.16 | 0.206 |
| Phosphate, mmol/L | 1.74 ± 0.38 | 1.70 ± 0.38 | 1.86 ± 0.37 | 0.101 |
| iPTH, pg/ml | 210.95 (124.47, 318.76) | 195.45 (119.40, 294.30) | 298.10 (187.73, 367.80) | 0.087 |
| Hemoglobin, g/L | 116.87 ± 7.32 | 116.57 ± 7.57 | 117.81 ± 6.59 | 0.506 |
| Albumin, g/L | 39.57 ± 2.15 | 39.78 ± 2.15 | 39.0 ± 2.09 | 0.130 |
| TG, mmol/L | 1.91 ± 1.15 | 1.90 ± 1.21 | 1.96 ± 0.93 | 0.815 |
| TC, mmol/L | 3.75 ± 0.88 | 3.72 ± 0.89 | 3.83 ± 0.89 | 0.626 |
| LDL-C, mmol/L | 1.92 ± 0.68 | 1.92 ± 0.71 | 1.95 ± 0.58 | 0.852 |
| CO_2_CP, % | 50.62 ± 5.34 | 50.42 ± 5.50 | 51.23 ± 4.91 | 0.550 |
| ALP, IU/L | 61.67 (51.88, 76.69) | 62.33 (52.92, 76.00) | 58.67 (49.79, 81.53) | 0.936 |
| Kt/V | 1.44 ± 0.28 | 1.41 ± 0.30 | 1.51 ± 0.23 | 0.195 |
| hsCRP, mg/L | 2.74 (1.05, 8.11) | 2.49 (0.96, 7.75) | 4.27 (1.24, 8.66) | 0.248 |
| Ferritin, ng/ml | 257.63 (217.10, 331.10) | 239.72 (211.46, 308.77) | 300.91 (258.30, 380.79) | **0.011** |
| 25-(OH)-D3, ng/ml | 16.06 ± 7.26 | 15.73 ± 6.36 | 17.06 ± 9.62 | 0.469 |

Abbreviations: BMI: body mass index; AAC score: abdominal aortic calcification score; DKD: diabetic kidney disease; CG: chronic glomerulonephritis; HRD: hypertensive renal damage; vBMD: volumetric bone mineral density; iPTH: intact parathyroid hormone; TG: triglyceride; TC: total cholesterol; LDL-C: low-density lipoprotein cholesterol; CO_2_CP: carbon dioxide combining power; ALP: alkaline phosphatase; hsCRP: high-sensitivity C-reactive protein; 25-(OH)-D3: 25-hydroxycholecalciferol

^a^ In this section, four patients were excluded because two of them had maintained hemodialysis for less than 6 months, and another two had been transferred from other hospitals to Beijing Jishuitan Hospital for less than 6 months

^b^ Because the sample size changed, the value corresponding to the 75th percentile of the AAC score (P75) changed to 122, which was used to divide the patients into the mild AAC group (n = 65) and severe AAC group (n = 21)

**^c^** Only the medications in the table were included in this study. Glucocorticoids, immunosuppressants, systemic anticoagulants and bisphosphonates that may affect AAC and vBMD were not included. The reason was that no patients received these medications during the same period of QCT.

**Supplemental Table 3** Association of AAC with continuous vBMD and vBMD stratification (the 6-month mean laboratory parameters)

| AAC degree | continuous vBMD | | |  |  | | | vBMD stratification | | | | | |
| --- | --- | --- | --- | --- | --- | --- | --- | --- | --- | --- | --- | --- | --- |
|  | B | OR (95% CI) | *P*-Values |  | Normal | Osteopenia | | | |  | Osteoporosis | | |
|  |  |  |  |  |  | B | OR (95% CI) | | *P*-Values |  | B | OR (95% CI) | *P*-Values |
| Model 1 | -0.025 | 0.976 (0.960, 0.992) | **0.004** |  | Ref. | 0.965 | 2.625 (0.610, 11.303) | | 0.195 |  | 1.956 | 7.071 (1.691, 29.565) | **0.007** |
| Model 2 | -0.026 | 0.974 (0.952, 0.996) | **0.022** |  | Ref. | 1.716 | 5.560 (0.906, 34.111) | | 0.064 |  | 2.338 | 10.360 (1.575, 68.171) | **0.015** |
| Model 3 | -0.032 | 0.969 (0.944, 0.994) | **0.017** |  | Ref. | 2.365 | 10.648 (1.318, 86.031) | | **0.026** |  | 2.760 | 15.796 (1.697, 147.052) | **0.015** |

Model 1 Unadjusted; Model 2 Adjusted for age, dialysis vintage and primary disease; Model 3 Adjusted for age, dialysis vintage and primary disease, phosphate, iPTH, Kt/V and ferritin

**2.2 Using other cutoff points of the AAC score**

In the main analyses, the 75th percentile (P75) of the AAC score was used as the cutoff point for the mild and severe groups, and then the association of AAC with continuous vBMD and vBMD stratification was analyzed. To examine the stability of the results, multivariate logistic regression models were carried out according to P70, P65 and P60 of the ACC score. Moreover, we also adopted the 6-month mean laboratory parameters as covariates in these logistic regression models to further examine the robustness of the results.

**Supplemental Table 4** Association of AAC with continuous vBMD and vBMD stratification (90 patients^a^, P70^b^)

| AAC degree | continuous vBMD | | |  |  | | | vBMD stratification | | | | | |
| --- | --- | --- | --- | --- | --- | --- | --- | --- | --- | --- | --- | --- | --- |
|  | B | OR (95% CI) | *P*-Values |  | Normal | Osteopenia | | | |  | Osteoporosis | | |
|  |  |  |  |  |  | B | OR (95% CI) | | *P*-Values |  | B | OR (95% CI) | *P*-Values |
| Model 1 | -0.023 | 0.978 (0.963, 0.992) | **0.002** |  | Ref. | 1.671 | 5.317 (1.314, 21.516) | | **0.019** |  | 2.195 | 8.976 (2.196, 36.697) | **0.002** |
| Model 2 | -0.023 | 0.977 (0.959, 0.996) | **0.016** |  | Ref. | 2.224 | 9.246 (1.729, 49.452) | | **0.009** |  | 2.425 | 11.300 (1.925, 66.318) | **0.007** |
| Model 3 | -0.026 | 0.975 (0.954, 0.995) | **0.017** |  | Ref. | 2.630 | 13.880 (2.069, 93.122) | | **0.007** |  | 2.606 | 13.550 (1.774, 103.490) | **0.012** |

Model 1 Unadjusted; Model 2 Adjusted for age, dialysis vintage and primary disease; Model 3 Adjusted for age, dialysis vintage and primary disease, phosphate, iPTH, Kt/V and ferritin

^a^ Data from the most recent laboratory parameters before QCT of 90 patients were used

^b^ The 70th percentile of the AAC score (P70) corresponding to 101.5 was used to divide the patients into a mild AAC group (n = 63) and a severe AAC group (n = 27)

**Supplemental Table 5** Association of AAC with continuous vBMD and vBMD stratification (90 patients^a^, P65^b^)

| AAC degree | continuous vBMD | | |  |  | | | vBMD stratification | | | | | |
| --- | --- | --- | --- | --- | --- | --- | --- | --- | --- | --- | --- | --- | --- |
|  | B | OR (95% CI) | *P*-Values |  | Normal | Osteopenia | | | |  | Osteoporosis | | |
|  |  |  |  |  |  | B | OR (95% CI) | | *P*-Values |  | B | OR (95% CI) | *P*-Values |
| Model 1 | -0.021 | 0.979 (0.966, 0.993) | **0.003** |  | Ref. | 1.227 | 3.411 (1.030, 11.290) | | **0.045** |  | 1.761 | 5.815 (1.722, 19.634) | **0.005** |
| Model 2 | -0.020 | 0.980 (0.963, 0.998) | **0.027** |  | Ref. | 1.629 | 5.101 (1.212, 21.474) | | **0.026** |  | 1.773 | 5.891 (1.249, 27.773) | **0.025** |
| Model 3 | -0.021 | 0.979 (0.961, 0.998) | **0.029** |  | Ref. | 1.827 | 6.216 (1.348, 28.654) | | **0.019** |  | 1.794 | 6.013 (1.137, 31.804) | **0.035** |

Model 1 Unadjusted; Model 2 Adjusted for age, dialysis vintage and primary disease; Model 3 Adjusted for age, dialysis vintage and primary disease, phosphate, iPTH, Kt/V and ferritin

^a^ Data from the most recent laboratory parameters before QCT of 90 patients were used

^b^ The 65th percentile of the AAC score (P65) corresponding to 93 was used to divide the patients into a mild AAC group (n = 59) and a severe AAC group (n = 31)

**Supplemental Table 6** Association of AAC with continuous vBMD and vBMD stratification (90 patients^a^, P60^b^)

| AAC degree | continuous vBMD | | |  |  | | | vBMD stratification | | | | | |
| --- | --- | --- | --- | --- | --- | --- | --- | --- | --- | --- | --- | --- | --- |
|  | B | OR (95% CI) | *P*-Values |  | Normal | Osteopenia | | | |  | Osteoporosis | | |
|  |  |  |  |  |  | B | OR (95% CI) | | *P*-Values |  | B | OR (95% CI) | *P*-Values |
| Model 1 | -0.019 | 0.981 (0.968, 0.994) | **0.003** |  | Ref. | 1.141 | 3.130 (1.002, 9.774) | | 0.050 |  | 1.689 | 5.417 (1.685, 17.417) | **0.005** |
| Model 2 | -0.018 | 0.982 (0.966, 0.998) | **0.031** |  | Ref. | 1.382 | 3.985 (1.080, 14.705) | | **0.038** |  | 1.673 | 5.331 (1.264, 22.473) | **0.023** |
| Model 3 | -0.018 | 0.982 (0.965, 0.999) | **0.039** |  | Ref. | 1.576 | 4.834 (1.193, 19.582) | | **0.027** |  | 1.581 | 4.862 (1.075, 21.988) | **0.040** |

Model 1 Unadjusted; Model 2 Adjusted for age, dialysis vintage and primary disease; Model 3 Adjusted for age, dialysis vintage and primary disease, phosphate, iPTH, Kt/V and ferritin

^a^ Data from the most recent laboratory parameters before QCT of 90 patients were used

^b^ The 60th percentile of the AAC score (P60) corresponding to 86 was used to divide the patients into a mild AAC group (n = 56) and a severe AAC group (n = 34)

**Supplemental Table 7** Association of AAC with continuous vBMD and vBMD stratification (86 patients^a^, P70^b^)

| AAC degree | continuous vBMD | | |  |  | | | vBMD stratification | | | | | |
| --- | --- | --- | --- | --- | --- | --- | --- | --- | --- | --- | --- | --- | --- |
|  | B | OR (95% CI) | *P*-Values |  | Normal | Osteopenia | | | |  | Osteoporosis | | |
|  |  |  |  |  |  | B | OR (95% CI) | | *P*-Values |  | B | OR (95% CI) | *P*-Values |
| Model 1 | -0.026 | 0.974 (0.959, 0.990) | **0.001** |  | Ref. | 1.303 | 3.682 (0.888, 15.274) | | 0.073 |  | 2.277 | 9.750 (2.339, 40.649) | **0.002** |
| Model 2 | -0.027 | 0.973 (0.953, 0.995) | **0.014** |  | Ref. | 1.915 | 6.786 (1.207, 38.138) | | **0.030** |  | 2.560 | 12.931 (2.028, 82.451) | **0.007** |
| Model 3 | -0.034 | 0.967 (0.942, 0.991) | **0.009** |  | Ref. | 2.653 | 14.191 (1.804, 111.629) | | **0.012** |  | 3.149 | 23.311 (2.497, 217.628) | **0.006** |

Model 1 Unadjusted; Model 2 Adjusted for age, dialysis vintage and primary disease; Model 3 Adjusted for age, dialysis vintage and primary disease, phosphate, iPTH, Kt/V and ferritin

^a^ Data from the 6-month mean laboratory parameters of 86 patients were used

^b^ The 70th percentile of the AAC score (P70) corresponding to 106 was used to divide the patients into a mild AAC group (n = 61) and a severe AAC group (n = 25)

**Supplemental Table 8** Association of AAC with continuous vBMD and vBMD stratification (86 patients^a^, P65^b^)

| AAC degree | continuous vBMD | | |  |  | | | vBMD stratification | | | | | |
| --- | --- | --- | --- | --- | --- | --- | --- | --- | --- | --- | --- | --- | --- |
|  | B | OR (95% CI) | *P*-Values |  | Normal | Osteopenia | | | |  | Osteoporosis | | |
|  |  |  |  |  |  | B | OR (95% CI) | | *P*-Values |  | B | OR (95% CI) | *P*-Values |
| Model 1 | -0.024 | 0.977 (0.962, 0.991) | **0.002** |  | Ref. | 1.274 | 3.575 (0.990, 12.913) | | 0.052 |  | 2.113 | 8.273 (2.219, 30.843) | **0.002** |
| Model 2 | -0.023 | 0.977 (0.959, 0.996) | **0.019** |  | Ref. | 1.655 | 5.233 (1.166, 23.495) | | **0.031** |  | 2.207 | 9.088 (1.759, 46.939) | **0.008** |
| Model 3 | -0.027 | 0.973 (0.953, 0.994) | **0.012** |  | Ref. | 2.169 | 8.750 (1.599, 47.867) | | **0.012** |  | 2.552 | 12.828 (2.022, 81.376) | **0.007** |

Model 1 Unadjusted; Model 2 Adjusted for age, dialysis vintage and primary disease; Model 3 Adjusted for age, dialysis vintage and primary disease, phosphate, iPTH, Kt/V and ferritin

^a^ Data from the 6-month mean laboratory parameters of 86 patients were used

^b^ The 65th percentile of the AAC score (P65) corresponding to 96 was used to divide the patients into a mild AAC group (n = 57) and a severe AAC group (n = 29)

**Supplemental Table 9** Association of AAC with continuous vBMD and vBMD stratification (86 patients^a^, P60^b^)

| AAC degree | continuous vBMD | | |  |  | | | vBMD stratification | | | | | |
| --- | --- | --- | --- | --- | --- | --- | --- | --- | --- | --- | --- | --- | --- |
|  | B | OR (95% CI) | *P*-Values |  | Normal | Osteopenia | | | |  | Osteoporosis | | |
|  |  |  |  |  |  | B | OR (95% CI) | | *P*-Values |  | B | OR (95% CI) | *P*-Values |
| Model 1 | -0.020 | 0.980 (0.967, 0.993) | **0.004** |  | Ref. | 1.061 | 2.889 (0.920, 9.071) | | 0.069 |  | 1.627 | 5.091 (1.544, 16.789) | **0.008** |
| Model 2 | -0.018 | 0.982 (0.966, 0.999) | **0.042** |  | Ref. | 1.342 | 3.826 (1.013, 14.453) | | **0.048** |  | 1.480 | 4.393 (1.012, 19.061) | **0.048** |
| Model 3 | -0.020 | 0.980 (0.962, 0.998) | **0.031** |  | Ref. | 1.725 | 5.611 (1.297, 24.277) | | **0.021** |  | 1.673 | 5.327 (1.099, 25.833) | **0.038** |

Model 1 Unadjusted; Model 2 Adjusted for age, dialysis vintage and primary disease; Model 3 Adjusted for age, dialysis vintage and primary disease, phosphate, iPTH, Kt/V and ferritin

^a^ Data from the 6-month mean laboratory parameters of 86 patients were used

^b^ The 60th percentile of the AAC score (P60) corresponding to 86 was used to divide the patients into a mild AAC group (n = 53) and a severe AAC group (n = 33)
